# Biological Characteristics of the MG-63 Human Osteosarcoma Cells on Composite Tantalum Carbide/Amorphous Carbon Films

**DOI:** 10.1371/journal.pone.0095590

**Published:** 2014-04-23

**Authors:** Yin-Yu Chang, Heng-Li Huang, Ya-Chi Chen, Jui-Ting Hsu, Tzong-Ming Shieh, Ming-Tzu Tsai

**Affiliations:** 1 Department of Mechanical and Computer-Aided Engineering, National Formosa University, Yunlin, Taiwan; 2 School of Dentistry, China Medical University, Taichung, Taiwan; 3 Department of Materials Science and Engineering, Mingdao University, Changhua, Taiwan; 4 Department of Dental Hygiene, China Medical University, Taichung, Taiwan; 5 Department of Biomedical Engineering, Hungkuang University, Taichung, Taiwan; University Hospital of the Albert-Ludwigs-University Freiburg, Germany

## Abstract

Tantalum (Ta) is a promising metal for biomedical implants or implant coating for orthopedic and dental applications because of its excellent corrosion resistance, fracture toughness, and biocompatibility. This study synthesizes biocompatible tantalum carbide (TaC) and TaC/amorphous carbon (a-C) coatings with different carbon contents by using a twin-gun magnetron sputtering system to improve their biological properties and explore potential surgical implant or device applications. The carbon content in the deposited coatings was regulated by controlling the magnetron power ratio of the pure graphite and Ta cathodes. The deposited TaC and TaC/a-C coatings exhibited better cell viability of human osteosarcoma cell line MG-63 than the uncoated Ti and Ta-coated samples. Inverted optical and confocal imaging was used to demonstrate the cell adhesion, distribution, and proliferation of each sample at different time points during the whole culture period. The results show that the TaC/a-C coating, which contained two metastable phases (TaC and a-C), was more biocompatible with MG-63 cells compared to the pure Ta coating. This suggests that the TaC/a-C coatings exhibit a better biocompatible performance for MG-63 cells, and they may improve implant osseointegration in clinics.

## Introduction

Tantalum (Ta) is a promising metal for use in biomedical implants or orthopedic and dental implant coatings because of its excellent corrosion resistance, fracture toughness, and biocompatibility [1]–[3]. Ta metal with its porous structure similar to that of spongious bone has been used in various orthopedic implants [4]–[7]. Studies [3], [8], [9] have shown that Ta metal is appropriate for osteogenesis in animal implantation tests, and in vivo studies have proven it suitable for cell adhesion, proliferation, and differentiation [10]–[12]. Ta has also been used to fabricate stents and artificial heart valves for cardiac and vascular devices because of its high corrosion resistance and radio-opacity properties [13], [14]. The biocompatibility of Ta have been identified and widely applied to various biomedical materials and devices.

Due to its biocompatibility, various Ta composites have been examined to improve physicochemical properties and interactions with cells in vitro and in vivo. Several Ta composites exhibit distinct material characteristics depending on the surface modification technique used and the chemical bonding structures [3], [6], [15]–[18]. Ta nitride (TaN) exhibits relatively high hardness, thermal stability, and superior corrosion resistance. It also produces extremely low friction against steel and alumina, and provides enhanced blood compatibility compared with TiN and Ta. A natural thin layer of tantalum oxide, which exists on the surface of Ta metal, might play a key role in its biocompatibility. Tantalum oxides and TaN are also compatible with cardiac and vascular devices.

An amorphous carbon (a-C) composite—because of its superior mechanochemical properties such as low friction coefficient, high hardness, and chemical inertness—is commonly used as a surface coating in medical devices [19]. Because of its excellent biocompatibility, a-C has been applied to the coatings of hip prostheses, orthopedic implants, vascular stents, and artificial heart valves [13], [20], [21]. A recent study showed that a-C coating on artificial hip joints reduced the wear debris [20]. Several in vitro studies have also demonstrated that a-C coatings improved the adhesion and growth of osteoblasts, reduced platelet attachment, and inhibited activation [22], [23].

In this study, TaC and TaC/a-C coatings with various carbon contents were synthesized using pulsed unbalanced magnetron sputtering in an argon (Ar) atmosphere, and were deposited onto glass cover slips. The cell viability of human osteosarcoma cell line MG-63 was evaluated. To observe and record the attachment and morphology of living cells in real-time using inverted optical and fluorescent confocal microscopy during the culture period, a glass cover slip was used as a substrate of Ta, TaC, and TaC/a-C coatings. Besides, the titanium (Ti) metal has been widely and clinically applied to implant materials and used as a substrate of various coatings in most cases, an uncoated Ti specimen with cultured cells was regarded as a control group for the comparison with these coatings in this study.

## Experimental Details

### 1.Sample preparation and characterization

Ta, TaC, and composite TaC/a-C coatings were deposited onto glass slides using unbalanced magnetron sputtering in an Ar atmosphere. High purity Ta and graphite targets (99.99 at.%) were arranged in conjunction to deposit the composite coatings. Each target had a diameter of 50 mm and was tilted 45° relative to the substrate. The target and substrate were separated by 60 mm, and the samples were placed on a rotational substrate holder for deposition. The base pressure before deposition was less than 2×10^−3^ Pa. Before deposition, the substrates were etched for 20 min at a substrate bias potential of −800 V in Ar plasma. The Ta, TaC, and composite TaC/a-C coatings were deposited by driving the cathodes in pulsed DC mode at a frequency of 20 kHz and a duty of 80% using Advanced Energy MDX power supplies with SPARCLE pulse units. To enhance film adhesion, Ta ion bombardment (Ta cathode power  = 50 W; bias voltage  = −600 V) was applied before deposition. After etching, the Ta coating was deposited, with the cathode power of the Ta target set to 200 W and a bias voltage of −40 V. Using a fixed Ar flow rate of 75 sccm, the chamber was maintained a deposition pressure of 0.6 Pa. To produce TaC and composite TaC/a-C that contained various carbon contents, the Ta cathode power was maintained at 20 W while the graphite cathode power was set to 150 W or 200 W. A Ta layer was deposited as an interlayer (approximately 80 nm). A substrate bias voltage of −40 V was used. The sample temperature during deposition was measured using a thermocouple located near the sample (range 100±25°C). The coating thickness was maintained at 0.3–0.6 µm by using a deposition time of 20 min.

The composition and chemical-binding characteristics of the deposited films were determined using X-ray photoelectron spectroscopy (XPS; PHI1600) with Mg Kα radiation. It was performed by sputtering the surface oxide layer with 2-kV Ar ions for 1 min to reveal the chemical composition of the deposited coatings. Survey spectra from 0 eV to 1000 eV were recorded for each sample, followed by high-resolution spectra for various elemental peaks, which were used to calculate the composition. Spectral ranges of 25±11 eV and 287±12 eV corresponded to Ta4f and C1s binding energies, respectively. The energy was calibrated by referring to the Au 4f_7/2_ peak from a clean gold surface at 83.8 eV. The surface morphology of the deposited coatings was examined using high-resolution field-emission scanning electron microscopy (SEM; JSM-7000F, Joel). Glancing-angle X-ray diffractometry (XRD; PANalytical X'pert Pro), at a glancing angle of 2°, and Cu radiation were employed for phase identification. The diffractometer was operated at 40 kV and 30 mA.

### 2.Contact angle measurement

The contact angle measurement was conducted after each specimen was cleaned using ultrasonic water bath for cell cultivation and in vitro biocompatibility analyses. The static contact angle of deionized water on each sample at room temperature was measured after the uncoated Ti and Ta, TaC and TaC/a-C-coated samples were alternately washed in ethanol and deionized water in an ultrasonic cleaner for 30 min. After the samples were dried in a clean dry oven at 55°C for 6 h, drops generated using a micrometric syringe were deposited onto the surface. The sample height-to-width ratio was measured and the samples were immediately photographed using an instrument to measure the contact angles (FTA-125, First Ten Angstroms, Portsmouth, VA, USA). Each contact angle reported herein is the mean of at least 10 independent measurements (n = 10 at least).

### 3.Cultivation of human osteosarcoma cell line MG-63

Human MG-63 osteoblast-like cells were purchased from Bioresource Collection and Research Center (product No. BCRC 60279, passage 101, BCRC, Hsinchu City, Taiwan). In this study, MG-63 cells were cultured in DMEM (Gibco, Carlsbad, CA) medium with 10% fetal bovine serum (Gibco) and 1% penicillin streptomycin (Gibco). Half medium was replaced every 2–3 days. After cells reached almost 90% confluences, cells were trypsinized and centrifuged as cell pellet. Cell pellets were resuspended in fresh medium for subculture. MG-63 cells at passages 103–105 were used in this study.

### 4.Biocompatibility tests of MG-63 cells

Biocompatibility was evaluated by assessing the cell viability of the MG-63 cells cultured onto the uncoated Ti, Ta-, TaC-, and TaC/a-C-coated samples. Cell viability was measured by detecting the intracellular purple formazan resulting from the yellow tetrazolium MTT (3-(4, 5-dimethylthiazolyl-2) -2, 5-diphenyltetrazolium bromide). MG-63 cells were seeded onto each specimen at a density of 5,000 cells/cm^2^ and incubated at 37°C in 5% CO_2_ for 5 days. An MTT (Sigma-Aldrich, St. Louis, MO) was added to the cultured cells and incubated for an additional 4 h. The purple formazan was eluted using 2 mL isopropanol-HCl (Sigma-Aldrich). The absorbance of the purple formazan was quantified as the optical density (OD) at 570 nm measured using a SpectraMax spectrophotometer (Molecular Devices, Sunnyvale, CA, USA) with SoftMax Pro 5.2 241 software (Molecular Devices). The OD of formazan reflects the level of cell metabolic activity. High OD values indicate correspondingly high cell viability. All procedures were protected from light irradiation and the experiments were independently duplicated (n = 6).

### 5.Cell morphology observation and fluorescent staining imaging

The cell morphology and attachment were observed and recorded using an inverted optical microscope (Olympus IX81) in a phase-contrast mode. Cells were observed daily before and after the medium was replaced. To locate and monitor the cell distribution, the nuclei were stained using Hoechst 33258 dye (Invitrogen, Carlsbad, CA). The distribution of cells with blue fluorescence-stained nuclei was observed and recorded using a spinning disk confocal microscope (Olympus CKX1). The cells were cultured onto each specimen at a relative lower density of 2,000 cells/cm^2^ for clear imaging collection. After 48-h incubation, the cells were washed twice in phosphate buffered saline (PBS) and fixed by immersing them in 3% paraformaldehyde for 10 min. Subsequently, 1 µg/mL of Hoechst 33258 dye was added and incubated with the cells for 15 min. After incubation, the cells were washed twice in PBS and each specimen was sealed to a glass slide by using nail oil. The fluorescent dye was excited at 350 nm and emitted at 460 nm. The blue fluorescence-stained nuclei were detected by using a DAPI filter.

### 6.Statistical analysis

The statistical correlation of the contact angle and the MTT test among the uncoated Ti, Ta-coated, TaC-coated and TaC/a-C-coated samples was determined using a Student's *t*-test. The sample size for the contact angle measurement was n = 10 at least. For MTT assay, the sample size was n = 6. Each experiment was independently performed and duplicated. The differences were considered significant at the *p*<.01 level.

## Results and Discussion

### 1. Microstructure analysis

**Figure 1:**
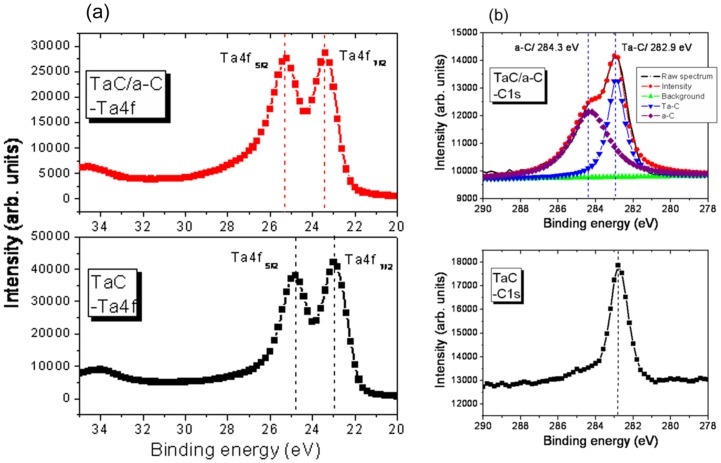
(a) Ta4f and (b) C1s XPS spectra of the deposited TaC and TaC/a-C coatings.

XPS was used to characterize the chemical composition and bonding states of the deposited TaC and TaC/a-C coatings. At a low graphite cathode power (150 W), the elemental composition of the TaC coating was 56.4±3 at.% Ta and 43.6±2.6 at.% C. As the graphite cathode power was increased from 150 W to 200 W, the carbon content increased to 56.5±2.5 at.% and the atomic content ratio of C/Ta increased from 0.8 to 1.3. High C/(Ta+C) cathode power ratios produced correspondingly high carbon content in the coatings. The TaC/a-C coating deposited at a graphite cathode power of 200 W exhibited the highest carbon content of 56.5±2.2 at.% with a Ta content of 43.5±2.8 at.%. Fig. 1 shows the Ta4f and C1s core level XPS spectra of the TaC and TaC/a-C coatings deposited at various graphite cathode powers. The coated samples are referred to as TaC and TaC/a-C at graphite cathode powers of 150 W and 200 W, respectively. The Ta4f spectrum (Fig. 1(a)) comprises both the Ta4f_7/2_ and Ta4f_5/2_ photoelectron lines with a 1.9-eV separation [24]. Regarding the TaC sample that exhibited a C content of 43.6 at.%, the Ta4f_7/2_ binding energy was 23 eV and the C1s was located at 282.9 eV. This is reasonably similar to the value that corresponds to the chemical bond between Ta and C [25], [26]. The Ta plasma reacted with C to form a TaC coating. As the C content increased to 56.5 at.%, the Ta4f_7/2_ peaks shifted to an increased binding of 23.4 eV for the TaC/a-C. This reflects an immediate chemical bonding change based on the variation in Ta and C contents, suggesting that more C incorporated with Ta to form TaC.

Previous studies have observed the presence of a-C in TiC/a-C:H and (Ti,Cr)(C,N)/a-C at a C content below 20 at.% [25], [27]. This study did not observe an obvious a-C peak in the TaC that exhibited a C content of 43.6 at.%. This suggests that C reacted with Ta to form TaC. As shown in Fig. 1(b), the TaC/a-C that exhibited a high C content of 56.5 at.%, showed a major pea in the C1s spectrum at 282.9 eV; this is characterized as Ta-C bonding. Another broad peak occurred at 284.3 eV, corresponding to a-C [28], [29]. The results show that the TaC/a-C coating comprised a mixture of TaC and a-C. The a-C bonding concentration can be derived from the C1s core level XPS spectra. The TaC/a-C contained 56.5 at.% a-C. Exceeding the C metastable solubility range within TaC, the C formed an a-C phase in the deposited coatings. This enabled the synthesis of TaC/a-C composite coatings.

**Figure 2:**
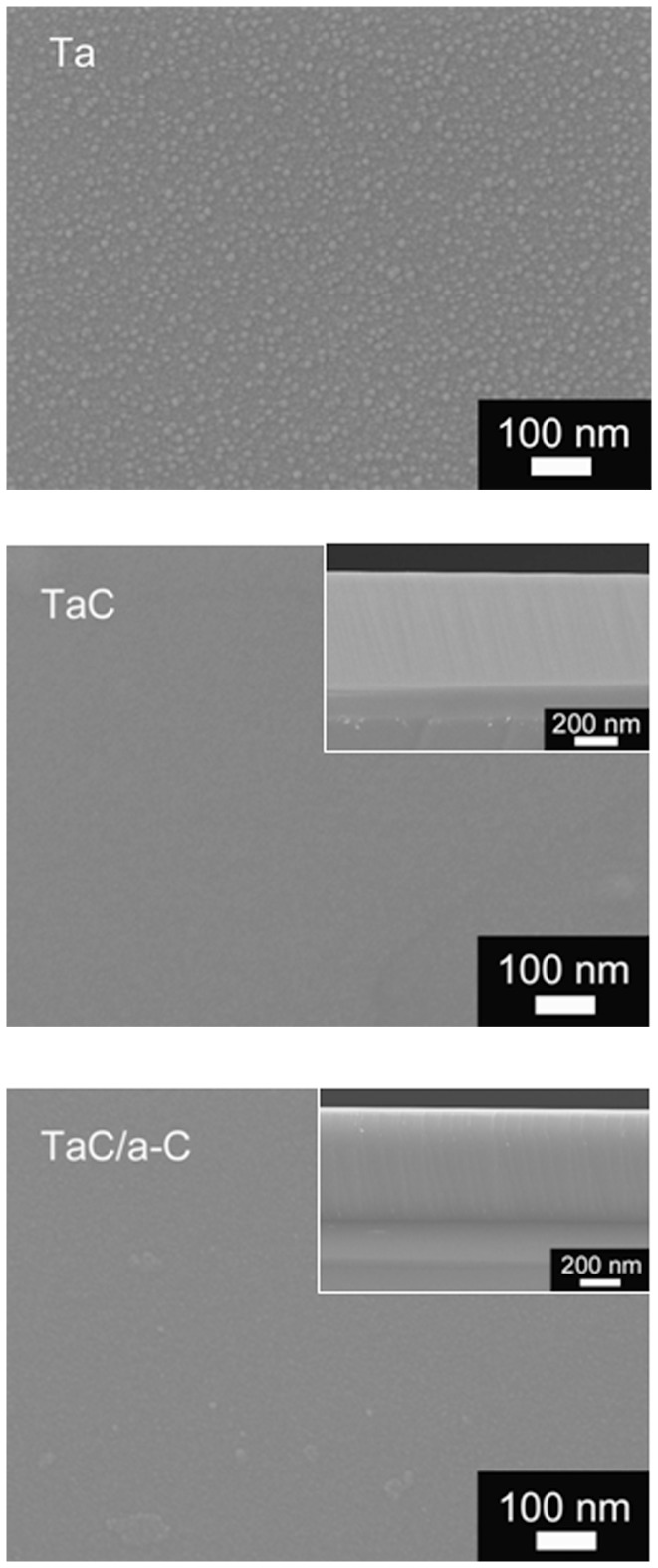
Surface SEM micrographs of the Ta, TaC, and TaC/a-C coatings.

**Figure 3:**
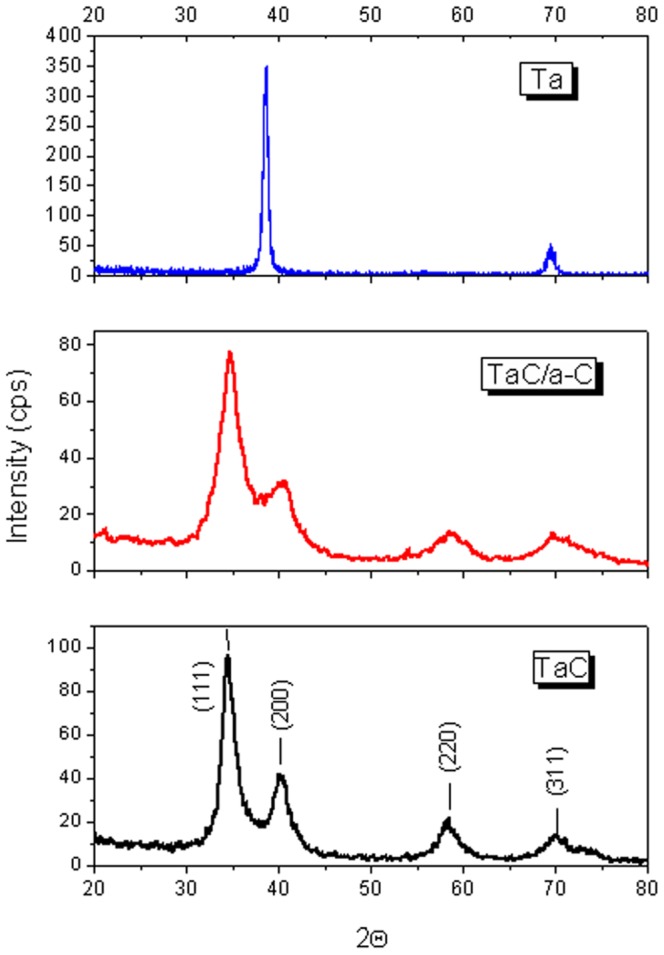
XRD spectra of the deposited Ta, TaC and TaC/a-C coatings.

Fig. 2 shows SEM micrographs of Ta, TaC, and TaC/a-C coatings. The TaC and TaC/a-C exhibit a columnar microstructure with a smooth surface. A compact microstructure evolved in the composite coating as the C content increased in the TiC/a-C:H and TiAl(C,N)/a-C coating systems [25], [30]. Fig. 3 shows typical XRD spectra from Ta, TaC and TaC/a-C samples with different carbon contents. Major peaks corresponding to the (111), (200), (220), and (311) planes for a NaCl-type crystalline structure were observed. The results reflect the presence of a cubic B1-NaCl structure in the deposited TaC (JCPDF file No.: #350801). The lattice parameter was 0.445 nm. The TaC/a-C coatings also exhibited a B1-NaCl crystal structure, demonstrating lower peak intensities, broader peaks, and finer grains than did the TaC. The intensity of the TaC reflections decreased as the C content increased, indicating that a small amount of C was in the a-C phase in the TaC/a-C coating and that the volume of crystalline TaC simultaneously decreased. Similar results were observed in the TiC/a-C coatings deposited using magnetron sputtering [31].

**Figure 4:**
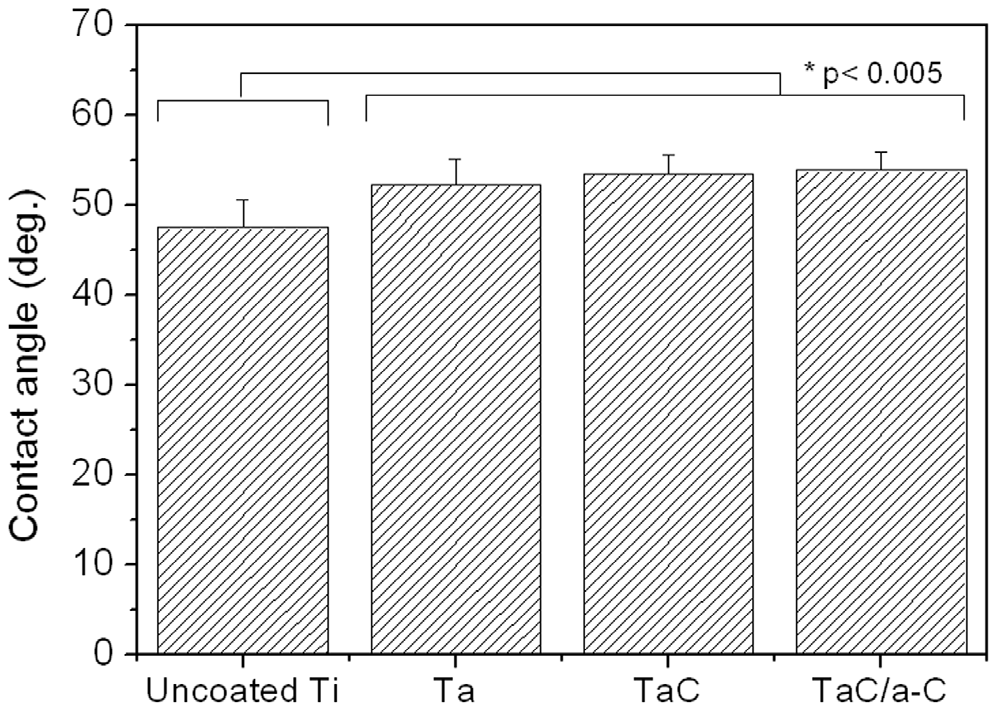
Contact angle values of uncoated, Ta-coated, TaC-coated, and TaC/a-C-coated samples. Data show the mean and SD values. Each specimen was n = 10 at least, and the experiment was performed duplicates. *Significantly different from the control group (*p*<.01).

To enhance bioactivity, the implants underwent surface modification to control properties such as their chemical nature and morphology. As shown in Fig. 4, the contact angle of the uncoated sample was 47.5°±3.1°. The Ta, TaC, and TaC/a-C-coated sample contact angles were 52.3°±2.9°, 53.6°±2°, and 53.9°±2.1°, respectively. The coated samples exhibited higher contact angles than did the uncoated samples (47.5°±3.1°; *p*<.005) and also showed hydrophobic properties on their surfaces. This finding is consisted with studies that have indicated that carbide and a-C films are hydrophobic and not porous, increasing the contact angle [32], [33].

### 2. Biocompatibility performance of Ta, TaC, and TaC/a-C coatings

**Figure 5:**
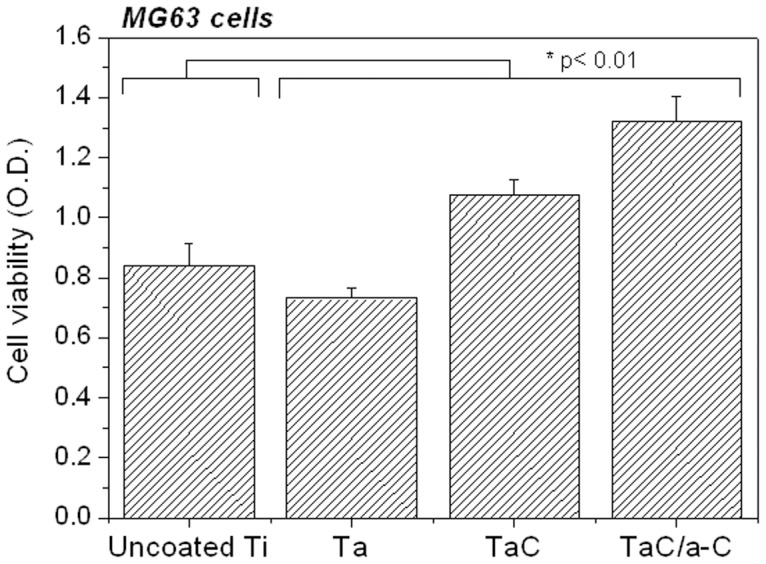
MTT assay test of MG-63 cells incubation with the uncoated, Ta-coated, TaC-coated, and TaC/a-C-coated samples at day 5. Data show the mean and SD values. Each specimen was n = 6, and the assay was performed duplicates. *Significantly different from the control group (*p*<.01).

Fig. 5 shows the absorbance of formazan (measured as OD by MTT assay) on MG-63 cells on the uncoated Ti and on the Ta-, TaC-, and TaC/a-C-coated samples. The TaC and TaC/a-C coatings exhibited higher cell viability than did the uncoated Ti and Ta-coated specimen, suggesting that TaC and composite TaC/a-C are the most biocompatible coatings. The TaC/a-C coating possessed the highest cell viability in the MG-63 cells. Given their excellent anticorrosion properties and biocompatibility, Ta metal and a-C are promising biomaterials for coating the surfaces of biomedical implants and devices. In most cases, the surface properties and long-term performance levels of biomaterials, particularly those applied to surgical implants and medical devices, are governed by surface modification techniques. Previous studies demonstrated that Ta composites improve cell viability and maintain the adherence, growth, shape and proliferation of cells [34]. This study successfully synthesized TaC and TaC/a-C coatings comprising various contents of carbon by using a twin-gun magnetron sputtering system. It revealed that the composite TaC/a-C coating, which contained two metastable phases (TaC and a-C), had the highest cell viability levels for MG-63 cells (Figs. 5).

Besides the contribution of TaC on cell viabilities, the improvement of biocompatibility of the TaC/a-C coating could be partially resulted from the a-C structure. Amorphous carbon has been a potential material used as a surface coating in medical devices because of its superior mechanochemical properties such as low friction coefficient, high hardness, and chemical inertness. Based on these advantages, an a-C film structure may also contribute to the high biocompatibility of cell attachment and proliferation. The biological effect of a-C coatings on various metal or polymer substrates exhibited good biocompatibility and haemocompatibility in different lineages of cells. The proliferation rates of smooth muscle and endothelial cells cultured onto a-C-coated stents were neither affected nor reduced [23]. The surface roughness of a-C increased the cell adhesion and proliferation of human fibroblasts [35].

**Figure 6:**
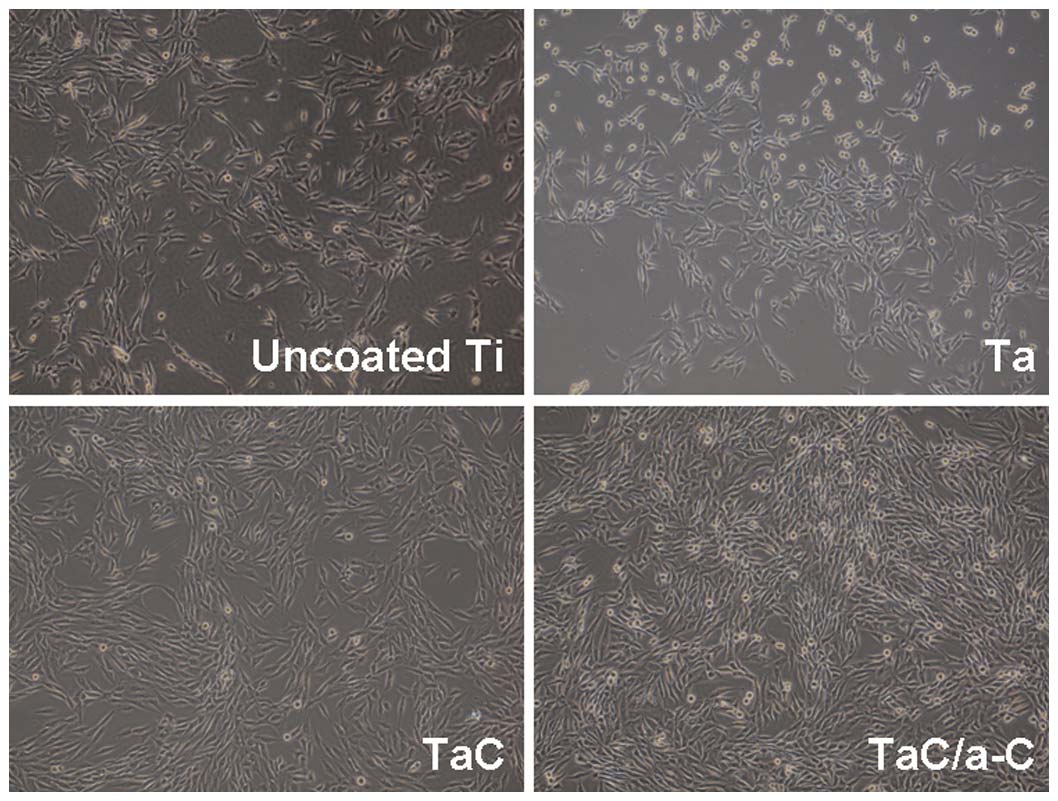
Morphology and distribution of MG-63 cells cultured onto uncoated, Ta-coated, TaC-coated, and TaC/a-C-coated samples for 72 h by using an inverted optical microscope. Cell density, 5000/cm^2^; original magnification, ×40.

**Figure 7:**
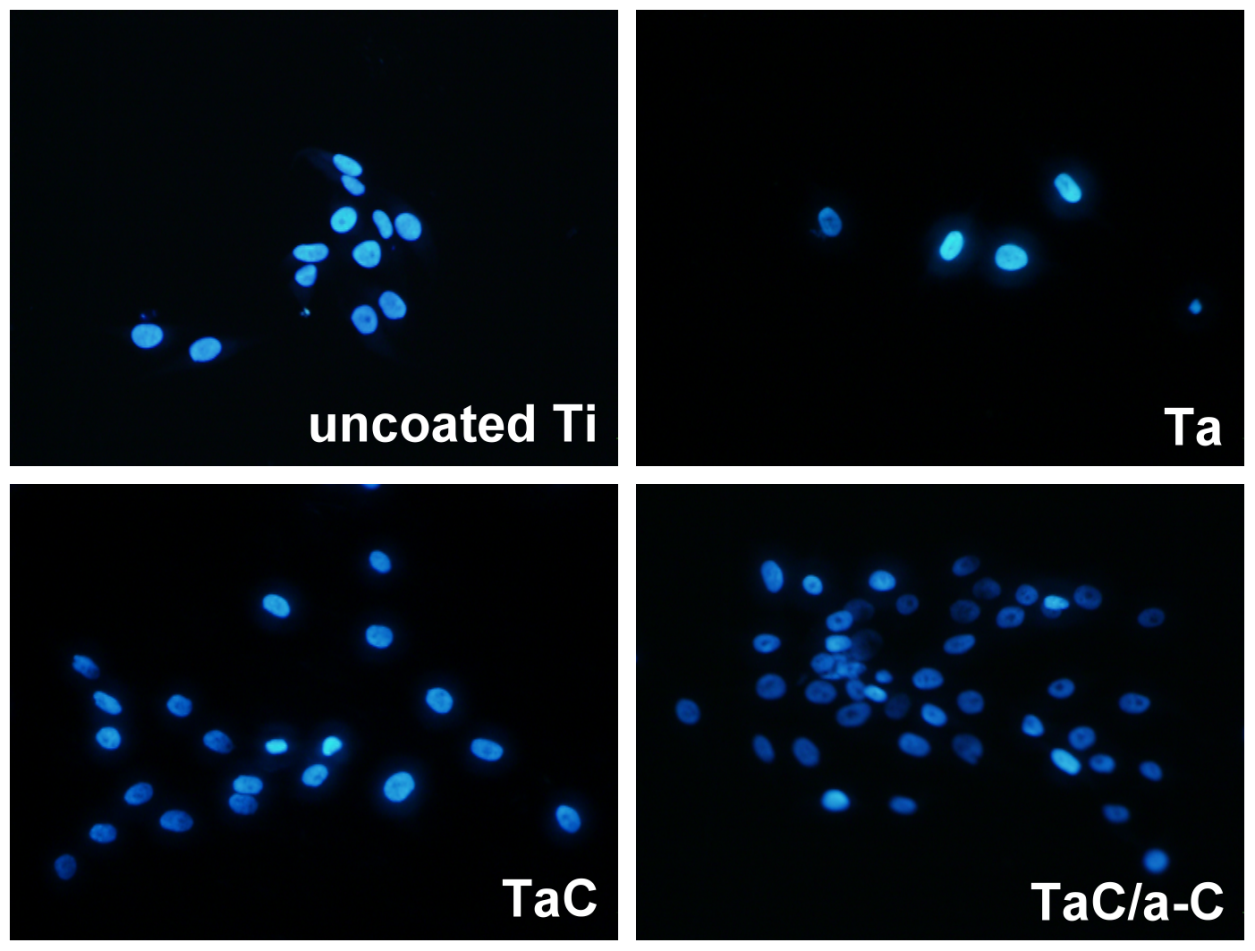
Spinning disk confocal imaging of MG-63 cells cultured onto (a) uncoated, (b) Ta-coated, (c) TaC-coated, (d) and TaC/a-C-coated samples at 48 h. The blue fluorescent-stained nucleus was stained using Hoechst 33258 dye. Cell density, 2,000 cells/cm^2^; original magnification, ×200.

Although SEM is the most common technique used to demonstrate the surface morphology of materials [44, 45], living cells cannot be easily monitored in real-time using SEM. In this study, a glass cover slip was used as a substrate for depositing thin films of Ta, TaC, and the TaC/a-C for light transmission. To observe the initial cell adhesion and cell proliferation during the whole culture period, an inverted optical microscope with a phase-contrast mode was used. To obtain clearer fluorescent imaging using a spinning disk confocal microscopy, MG-63 cells with cell density of 2,000 cells/cm^2^ were cultured on the uncoated Ti, Ta, TaC-, and TaC/a-C-coated films. Figure 6 and 7 showed the results of the cell distribution of MG-63 cells with higher and lower cell densities on each sample at 72 h and 48 h using inverted optical microscopy and fluorescent confocal microscopy, respectively. It provided a real-time observation of living cells during the culture period, demonstrating that a thin film on a glass cover slip was successful for optical and fluorescent microscopy imaging. It suggested that the labeling and tracking of organelles, proteins, or molecules in cells on a metal or metal composite thin-film by using glass as a substrate could be proceeded. Although the coated specimens showed more hydrophobic properties than did the uncoated sample, cell attachments occurred in the first 2 h after cells seeded onto each specimen. The overall attachment was completed in 6 h (data not shown). No obvious cell morphology differences were observed during the culture period (Fig. 6).

Fluorescent confocal imaging was used to show the adhesion and distribution of MG-63 cells with blue fluorescent-stained nuclei cultured onto each sample (Fig. 7). To detect any possible nuclear damage gradation and display the distribution of MG-63 cells on each coating, Hoechst 33258, a blue fluorescent dye sensitive to DNA conformation was used to bind with DNA located in the nucleus [36]. No DNA damage (e.g., DNA ladders or fragments) was detected in the specimens. The cell distribution of each group at 48 h was similar to the cell viability results at day 5. This indicated that the composite TaC/a-C was the most available and suitable coating for cell attachment and distribution. Several techniques can be used to produce a-C coatings that demonstrate proven biological effects, including plasma immersion ion implantation and deposition, magnetron sputtering, ion beam deposition, radio frequency plasma enhanced chemical vapor deposition, and a filtered cathodic vacuum arc. This study suggests that a composite TaC/a-C coating deposited by unbalanced magnetron sputtering may be an alternative to current materials used in biomedical applications.

## Conclusion

In this study, Ta, TaC, and composite TaC/a-C coatings were synthesized using twin-gun unbalanced magnetron sputtering. After controlling the pure graphite and Ta cathode powers, Ta plasma reacted with C to form a TaC coating. The results of XPS analyses show that it is possible to synthesize a composite TaC/a-C coating comprising two metastable phases (TaC and a-C). When the metastable solubility range of C was exceeded within the tantalum carbide, the C formed an a-C phase in the deposited coatings.

Cell viability analyses showed that TaC and composite TaC/a-C coatings possessed good biocompatibility for MG-63 cells. The optical and fluorescent confocal imaging also consistently demonstrated the cell attachment and distribution of MG-63 cells were similar to the results of cell viability. It suggested that the composite TaC/a-C coating exhibited the highest biocompatibility for cell adhesion, distribution, and growth. These findings indicated that the composite of TaC with a-C structure (TaC/a-C) was highly beneficial in improving the in vitro biocompatibility for MG-63 cells.
